# Supplementary material for: Are carbon emissions trading and green financial instruments synergistic? -Comprehensive quantitative research based on content analysis
Source: PLoS One. 2024 Mar 7;19(3):e0298601. doi: 10.1371/journal.pone.0298601 (PMC10919601; doi:10.1371/journal.pone.0298601)
Supplement: S1 Data — (ZIP) [file pone.0298601.s001.zip › Data/地级市Z_F工作报告.zip!/╣≤╓▌/╫±╥σ╩╨/└┤╫╘╣1⁄2╓┌║┼í╛┬φ┐╦╩2╛▌═°í┐_2015.docx]

各位代表：

现在，我代表市人民政府向大会作工作报告，请予审议，并请列席人员提出意见。

2014年工作回顾

过去一年，在省委、省政府和市委的正确领导下，在市人大、市政协的监督和支持下，我们认真贯彻落实习近平总书记系列重要讲话精神和中央、省、市各项决策部署，凝心聚力、克难攻坚、砥砺奋进，全市经济社会发展取得新成就，改革发展稳定各项工作开创新局面，为全面完成“十二五”规划目标、加速全面小康进程奠定了坚实基础。

后发赶超步伐加快。综合经济实力显著增强，工业经济总量在全省扩大领先，社会消费品零售总额增速在全省领跑，经济发展群众满意度测评全省第一，增比进位综合排名全省第二。城镇化率、财政收入、进出口总额等6项指标提前实现“十二五”规划目标。经济总量有望跻身西部30个同类城市前五位。

深化改革开局良好。转变政府职能，为市场松绑，给企业添力。重点推进“十项改革”，完成了市、县政府机构改革等43项改革任务，市级非行政许可事项全部取消，生态文明建设体制机制改革全省领先，公共资源交易平台改革走在全国前列。

人民生活日益改善。投资109亿元完成“民生十件实事”。21万农村贫困人口和25个贫困乡镇实现脱贫。高桥、董公寺、忠庄高速公路入城口和昆明路、官井隧道复线等交通“肠梗阻”得到疏解，问题楼盘、廉租房等关系群众切身利益的问题得到妥善处理，入学难、就医难等老百姓关注的民生难题逐步化解。

干部作风明显转变。扎实开展党的群众路线教育实践等系列活动，严格执行中央、省、市作风建设各项规定，坚持“五梳理三带头”，干部作风更加求真务实，干事创业激情更加高涨，形成了风清气正、心齐气顺的发展环境和上下一心、干群同心的强大合力，保障了各项工作高效推进，夺取了抗击“8.11”特大洪灾等重大胜利。

遵义形象大幅提升。纪念遵义会议80周年系列庆祝活动及茶博会、书博会、旅发大会等活动的成功举办，展示了我市经济社会发展所取得的辉煌成就，传递了全市人民共筑“遵义.中国梦”的美好愿景，提升了“红色圣地.醉美遵义”的知名度和美誉度。遵义会议精神激励我们更加奋勇前行！

一年来，我们主要抓了以下工作。

一、实施主基调主战略（1），开创经济发展新局面

据初步统计，全年完成地区生产总值1874.36亿元，同比增长（下同）14.4%。三次产业结构为14.3：45.9：39.8。500万元以上规模工业增加值872.7亿元，增长17.4%；2000万元以上规模工业增加值818.9亿元，增长12.4%。50万元以上固定资产投资2052.3亿元，增长31%。财政总收入412.66亿元，增长15.7%；一般公共预算收入159.65亿元，增长16.7%。社会消费品零售总额533.4亿元，增长13.4%。城镇居民人均可支配收入22728元，增长9.5%；农村居民人均可支配收入8365元，增长12.7%。年末金融机构存款、贷款余额分别为2567.6亿元、1527.7亿元，分别增长15.5%、26.9%。全面小康实现程度达83%。

工业经济在转型升级中排难前行。坚持全神贯注抓园区，凝神聚气抓工业。扎实推进工业“双服务”行动（2）和“百千万”工程（3），帮扶企业1966户；出台扶持工业发展政策措施，力保工业平稳运行。完成工业投资920亿元，建成以晴光电、桐梓电厂、肯富来等项目111个，建成标准厂房153万平方米，新增规模以上工业企业219家，实现信息产业产值410亿元、白酒产量40万千升。煤矿兼并重组扎实推进，关闭小煤矿50对，实现煤炭产量1200万吨；风电、页岩气等新能源开发进展顺利。

城镇建设在统筹规划中快速推进。启动“黔北城市群”规划，中心城区200万人口城市框架基本形成，完成城建投资1220亿元，城镇化率达43%。开展城乡规划“多规融合”试点，深入实施城市倍增计划，中心城区、各县（市）城市面貌和综合承载能力大幅提升。21个省级城市综合体和54个市级以上示范小城镇建设加快推进。

农业生产在结构调整中丰产增收。大力实施“1234”战略（4）和“8531”工程（5），实现粮食产量291.6万吨，完成农业增加值267.5亿元，规模农产品加工业产值达254亿元。35个省级现代高效农业示范园区实现销售收入80亿元。市级以上龙头企业累计415家、农民合作社2696个。绥阳“五朵金花”等“四在农家.美丽乡村”精品示范点吸引省内外游客慕名前往，探索出农旅融合发展新路子。

服务业在做实基础中加快发展。新增3个4A级景区，建成8家四星级以上酒店，接待游客5176.3万人次，旅游总收入444.4亿元，分别增长20.3%、24.5%。遵义国际商贸城、新雪域等大型商贸、物流项目投入运营，公路货运、客运周转量分别增长74.3%、64.3%。“引金入遵”金融机构4家。发行企业债券60亿元。“阿里巴巴.遵义产业带”、爱特购等电商平台加快发展，1500余种商品实现网上交易。新增限额以上商业企业138家。

二、统筹区域协调发展，展现区域发展新气象

中部率先崛起基础夯实。中部六县（区）狠抓项目建设，预计完成地区生产总值972.3亿元、固定资产投资1093.3亿元、工业增加值292.4亿元，分别占全市的50.8%、53.3%、35.7%。红花岗区在全市率先实现全面小康。汇川区持续优化经济结构，发展环境明显改善。新蒲新区统筹推进战略性新兴产业发展和新区建设，产城互动、校城互动取得新成效。遵义县全面统筹城乡发展，农村环境综合整治等多项工作全省领先。桐梓县发挥遵渝合作“桥头堡”作用，加快高新技术产业和乡村旅游发展。绥阳县依托资源环境做文章，引进建成一批新企业，综合考核名列全省非经济强县第一。

西部全域突破势头强劲。西部三县（市）围绕“四河四带”（6）建设，加速推进茅台酒“十二五”技改、茅台示范小城镇等项目，预计完成地区生产总值626.9亿元、固定资产投资554.7亿元、工业增加值478.1亿元，分别占全市的32.8%、27%、58.4%。仁怀市跻身全国百强县（市）和最具投资潜力百强县（市）。赤水市加快国际旅游目的地建设，旅游人次和综合收入均创历史新高。习水县加快推进能矿产业升级，建成一批安全高效现代化矿井，引进30多亿元推进习部生态改革试验区建设。

东部联动开发格局形成。东部三县围绕生态文明示范区建设，加快生态产业发展，湄潭翠芽、凤冈锌硒茶、“茅贡大粒香”等农产品品牌影响力不断扩大。预计完成地区生产总值171亿元、固定资产投资224亿元、工业增加值35.5亿元，分别增长17%、29.8%、17.4%。湄潭县大力推进茶旅一体化，茶业综合效益位居全国第二。凤冈县成功创建国家级出口食品农产品质量安全示范区。余庆县打造全景域生态旅游城市取得积极进展，致力打造旅居农家最美乡村，城乡统筹步伐加快。

北部全速攻坚成效明显。北部三县围绕扶贫攻坚主要任务，实现8.4万人脱贫，占全市的40%，务川自治县成功“减贫摘帽”。预计完成地区生产总值142.6亿元、固定资产投资180.2亿元、工业增加值12.9亿元，分别增长17.2%、28.5%、18.3%。正安县立足返乡农民工创业引进乐器制造等产业，立足加快城市发展打通凤仪至安场城市快速通道。道真自治县突破工业和基础设施瓶颈，建设园区标准厂房筑巢引凤。务川自治县煤电铝项目积极推进，成功承办第十一届中国羊产业发展大会，荣获“全国民族团结进步模范集体”称号。

三、推进改革开放创新，激发转型跨越新活力

重点改革扎实推进。启动了首批55项改革，乡镇行政管理体制改革试点全面启动，建立落实并联审批制度，部门权力清单、服务清单、责任清单实现“三单合一”。财税体制改革积极推进，“营改增”试点范围扩大，落实国家系列减免税政策，全年减免税收75亿元左右。工商登记制度改革有力推进，实行注册登记“先照后证”（7）。湄潭县获批全国第二批农村改革试验区。深化医药卫生体制改革扎实推进。投融资体制、国有企业产权制度、资源配置体制、社会事业等重点改革有序推进。

开放合作全面深化。上海对口帮扶深入推进，上海漕河泾新兴技术开发区遵义分区、遵义（上海）产业园建设加快。渝遵战略合作不断深化，与贵阳、泸州等地区和清华同方、上海延华等企业建立战略合作伙伴关系。遵义机场航空口岸已获批准，遵义综合保税区申建取得阶段性进展，设立了3个保税仓。新增外贸企业86家，实现外贸进出口总额4.6亿美元，增长22%；实际利用外资1.3亿美元，增长30%。引进500强企业10家，省外到位资金达1580亿元，增长35%。

发展活力不断增强。扎实推进国家创新型试点城市建设，市科技创新公共服务中心建成投用，新增院士工作站3个、高新技术企业10家、科技企业孵化器4家；获省科技奖13项，专利申请2800件；综合科技进步水平指数全省第二。引进高层次人才501名、各类急需紧缺人才8458名。扶持发展微型企业1939户，民营经济占地区生产总值比重达52.5%。培育19户企业分别在上海和贵州股权交易中心挂牌。

四、突出生态文明建设，彰显持续发展新理念

“创模”攻坚目标实现。成功创建国家环境保护模范城市。实施“天蓝、地绿、水清、气净、城乡清洁”工程，八大类223项环保基础设施日益完善，环保能力稳步提升，环境质量明显改善，群众实实在在感受到“创模”带来的好处。城市（县城）环境空气质量达标率95.4%，集中式饮用水源地水质达标率100%。

生态建设成效明显。各项生态指标位居全省前列。完成营造林75万亩，石漠化治理173.5平方公里，森林覆盖率达53.9%。中心城区绿地率、绿化覆盖率、人均公共绿地面积均列全省第一。“四河四带”生态文明先行示范区建设扎实推进，《赤水河流域生态补偿暂行办法》等6个生态文明建设制度（方案）上升为省级层面制度设计。5个湿地通过国家林业局评估。累计创建国家级生态示范区5个、生态镇39个，省级生态县3个、生态乡镇24个、生态村45个。

资源环境保护加强。严守耕地红线，完成中心城区周边和5000亩以上坝区永久基本农田划定工作，开展土地增减挂钩和耕作层剥离再利用，强化地质灾害防治。新建各类污水处理厂12座，中心城区生活垃圾利用水泥窑协同处置项目、4座大型压缩式垃圾中转库建成投用，城镇污水处理率83.8%，城乡生活垃圾无害化处理率77%。关闭淘汰落后产能11户118.2万吨，单位生产总值能耗下降4%，主要污染物排放总量控制在省下达指标内。

五、狠抓基础设施建设，增强加快发展新动力

交通条件明显改善。完成交通投资240亿元。新开工4条高速公路145公里，北环、余凯等高速建成通车，通车总里程突破700公里；改造国省干道109公里；建成通村油路1537公里。遵义机场通航城市达19个，旅客吞吐量67万人次；仁怀机场获国家批准启动建设。川黔铁路外迁正式获批，渝黔快铁全面开工，阁老坝铁路货场建设有序推进，城市轨道交通完成线路规划编制。乌江航运196公里四级航道、4个500吨级码头建成投用。

水电讯设施明显改善。完成水利投资51.8亿元。实施骨干水源工程34个，中桥、新桥等5座水库下闸蓄水，新增供水能力2亿立方米，解决80.8万农村人口饮水安全问题。新增有效灌溉面积16.8万亩。完成电力投资12亿元，改造35千伏及以下农村电网380公里，农村电网供电可靠性大幅提升。完成通讯投资23亿元，在全省率先实现移动4G信号乡乡通，与贵阳、安顺实现通讯同城化。

城乡面貌明显改善。“八大工程”（8）开工项目160个，累计投资332.8亿元。实施中心城区绿化、亮化、美化、净化工程，遵义会议陈列馆改扩建、城乡规划展览馆等项目建成开放，68座环卫公厕免费投用，中心城区供水实现互联互通；建成公交专用道、港湾式公交停靠站等配套设施42个，城市交通拥堵有效改善，城市环境明显提升。“四在农家.美丽乡村”升级工程“6+1”行动计划（9）全面推进，33个市级精品示范点成效明显。

六、着力保障和改善民生，取得全面小康新进展

社会事业发展加快。深入实施教育“9+3”行动计划，在全省率先实施基本普及十五年教育，学前三年毛入园率82%，9年义务教育巩固率89.5%，高中阶段毛入学率82%，中职毕业生就业率98%。遵义大学城完成投资41.5亿元，遵义职院、遵义医学院、遵义医专先后入驻；遵义大学申建进展顺利。实施教育工程1592个，中心城区新（改、扩）建中小学校7所。海龙屯申遗经过世界遗产委员会专家现场评审。公共文化服务体系示范区建设有序推进，遵义红色文化产业园获“国家级文化产业示范基地”称号。建成县级综合档案馆6个。全面实行单独二孩生育政策，人口出生率、自增率分别为10.27‰、4.89‰。遵义第七次荣获“全国无偿献血先进城市”。巩固国家卫生城市长效管理机制基本建立，仁怀、湄潭、道真创建国家卫生城市（县城）通过暗访和技术评估。食品药品安全、卫生监督、疾病防控、妇幼保健、民族宗教、妇女儿童、老年人、残疾人、人民防空、国防教育、国防后备力量建设、气象水文、地方志、红十字等工作全面推进。

社会保障能力提升。城镇新增就业11.4万人，转移农村劳动力9万人，城镇登记失业率2.8%。城乡低保标准分别提高13.1%、16.5%。新农合参合率98.2%，全年兑现补偿金23.5亿元。启动城镇居民大病保险工作，成功实现省内异地就医实时联网结算。新(改)建农村敬老院65所，实现乡镇全覆盖。建成城镇保障性住房20490套，改造农村危房42500户，扶贫生态移民4534户20608人。162.8万户农房纳入政策性灾害保险。

社会治理全面进步。“平安遵义”建设深入开展，严厉打击各类违法犯罪。全面推广社会管理“余庆经验”，加强重大事项社会稳定风险评估，建立诉访分离和领导包案督访、包案下访、包案化解等工作机制。没有发生重特大生产安全事故、重大群体性事件和重大刑事案件，生产安全事故起数和死亡人数持续“双降”；人民群众安全感、满意度分别达97.48%、97.03%，均排全省第二。

过去一年，我们认真践行党的群众路线，全面加强作风建设，强化行政监察、审计监督和督查督办，查处违纪违法案件867件、党政纪处分976人，有效整治了一批“四风”突出问题。组建244个民生项目监督特派组，办结3529个民生政策不落实问题，涉及金额5855.2万元；审计核减工程投资额8.67亿元。坚持依法行政、政务公开和厉行节约，全市党政机关“三公经费”支出同比下降27.6%。定期向市委、市人大报告工作，向市政协通报情况，自觉接受人大法律监督、政协民主监督和社会舆论监督，加强“市长-群众交流直通平台”建设，办结人大代表建议154件、政协委员提案422件，满意率和基本满意率达99%以上。

各位代表！这些成绩的取得，是省委、省政府和市委正确领导的结果，是市人大、市政协监督和支持的结果，是全市广大干部群众团结奋斗和各方大力支持的结果！在此，我代表市人民政府，向全市人民，向人大代表、政协委员，向各民主党派、工商联、无党派人士、人民团体，向驻遵解放军、武警消防官兵和民兵预备役、人民警察，向历届老领导老同志，向所有关心支持参与遵义发展的各界人士，表示衷心的感谢！

各位代表！过去一年的实践证明，面对新常态、新形势和新情况，我们必须始终保持政治定力，坚持党的领导不动摇，深入学习贯彻党的十八大、十八届三中、四中全会精神和习近平总书记系列重要讲话精神，自觉在思想上、政治上、行动上与党中央保持高度一致；必须始终保持战略定力，坚守两条底线不动摇，坚定不移实施主基调主战略，正视外部环境的复杂性，把中央和省委、省政府、市委各项决策部署落到实处；必须始终保持改革定力，坚持用好改革关键一招不动摇，充分发挥市场在资源配置中的决定性作用，不断破除影响经济社会发展的体制机制障碍；必须始终保持发展定力，坚持后发赶超、全面小康目标不动摇，统筹好改革发展稳定和改善民生各项工作，凝聚“两加一推”正能量，奋力推进经济平稳增长、民生持续改善、社会和谐稳定。

各位代表！在充分肯定成绩的同时，我们也清醒地看到存在的主要困难和问题，主要是：经济总量小，人均水平低，增收难度大，民生保障任务繁重，扶贫攻坚任务艰巨；经济下行压力持续加大，产业结构矛盾突出，项目融资瓶颈凸显，市场消费预期不稳，部分要素保障不足，外向型经济发展滞后，稳增长、调结构、转方式的压力大；全面深化改革进入深水区和攻坚期，体制机制性矛盾突出，开放水平不高，创新能力不强，人才资源匮乏，防范、化解和管控各类风险的压力大；政府效能亟待提高，干部作风亟待改进，发展环境亟待优化，转变政府职能、整治作风顽疾依然任重道远。这些都需要我们在发展中采取更加有力的措施加以解决。

2015年工作总体要求和目标重点

2015年是全面深化改革的关键之年，是全面推进依法治市的开局之年，也是全面完成“十二五”规划的收官之年。抓好2015年各项工作，机遇与挑战并存，动力与压力同在。稳中求进是当前经济工作总基调，中央和省稳增长调结构的宏观政策取向利好，全面深化改革的政策效应和改革红利正逐步释放，新一轮对外开放、基础设施建设和生态环境后发优势将带来更多新机遇，全市经济结构趋优、活力彰显、后劲增强，具备经济平稳较快增长的有利条件。新形势下，我们必须坚定信心、抢抓机遇、迎难而上，更加有为做好2015年各项工作。

2015年政府工作的总体要求：深入贯彻党的十八大和十八届三中、四中全会精神，以邓小平理论、“三个代表”重要思想、科学发展观为指导，深入贯彻落实习近平总书记系列重要讲话精神和中央、省、市经济工作会议精神，坚持稳中求进工作总基调，坚持以提高经济发展质量和效益为中心，坚持守住发展和生态两条底线，主动适应经济发展新常态，深入实施主基调主战略。着力“三化”互动、区域联动、投资拉动、开放带动、创新驱动、民生推动，实现总量大、质量好、均量高，防范和化解各类风险，保持经济持续较快发展和社会和谐稳定，确保全面完成和超额完成“十二五”规划目标任务。

2015年经济社会发展的主要预期目标：地区生产总值增长13.5%以上，达2180亿元。三次产业结构调整为13.3:46.3:40.4。2000万元以上规模工业增加值增长13.5%以上，达930亿元。50万元以上固定资产投资增长20%以上，达2500亿元以上。社会消费品零售总额增长14%以上，达608亿。财政总收入增长10%，达455亿元；一般公共预算收入增长13%，达180亿元。城镇居民人均可支配收入增长12%以上，达25530元；农村居民人均可支配收入增长14%以上，达9552元，力争突破1万元。全面小康实现程度86%以上。城镇登记失业率控制在4.2%以内。人口出生率、自增率控制在10.55‰、5.05‰以内。居民消费价格指数控制在103%左右。节能减排、安全生产指标控制在省下达目标范围以内。

各位代表！经济发展步入新常态，抓好2015年工作意义尤为重要。面对新形势新任务新要求，我们必须正确认识新常态、主动适应新常态、科学引领新常态，更加注重工作方法的针对性，更加注重工作举措的有效性，更加注重工作落实的实效性，突出“赶、转、改、创、治”五个重点，统筹推进“十项改革”、实施“十大工程”、办好“十件民生实事”。

突出“五个重点”。一是赶，就是全力稳增长。坚持发展第一要务，牢牢守住发展底线，保持经济在高于全国全省的合理区间增长。决战决胜“十二五”，谋划编制好“十三五”，奠定率先全面小康坚实基础。二是转，就是加快转方式。把转方式调结构放在更加重要的位置，牢牢守住生态底线，加快产业转型升级，调整投资方式和结构，优化区域空间布局，充分挖掘消费潜力，加速外向型经济发展，提高经济发展质量和效益。三是改，就是狠抓改革攻坚。坚持用好改革关键一招，以改革统揽发展全局，以改革破解发展难题，以改革增强内生动力，以改革增进人民福祉，以改革营造开放环境，以改革提升政府效能。四是创，就是突出创新驱动。把创新贯穿于经济社会发展各领域各环节，把创新落实到创造新的增长点上，一手抓制度创新，一手抓科技创新，让创新成为驱动发展的新引擎。五是治，就是建设法治政府。坚持法定职责必须为、法无授权不可为。努力建设职能科学、权责法定、执法严明、公开透明、廉洁高效、守法诚信的法治政府，以政府依法行政带动全民依法办事，以全民依法办事促进政府依法行政。

推进“十项改革”。一是深化行政管理体制和行政审批制度改革。进一步转变政府职能，推行政府向社会力量购买服务，完成机关公务用车制度改革；进一步扩权强县强镇，放活企业自主经营权。二是深化统筹城乡综合配套改革。积极推进湄潭县全国第二批农村改革试验区和全省第五轮农村综合改革试点。抓好农村土地承包经营权确权登记颁证，推进农村产权交易市场建设。三是深化投融资体制和财税体制改革。加强信用、担保体系建设，健全政银企沟通协调机制；创新投融资合作方式，促进政府与社会资本合作（PPP）。认真贯彻执行新《预算法》，深化财税体制改革，扩大“营改增”试点范围，完善预算管理制度。加强政府性债务管理，健全政府性债务风险防控机制，研究制定地方政府性债务管理办法。四是深化新型城镇化管理体制改革。加快中心城区城市管理体制改革，促进财权、事权、责权相统一；积极推进遵义县、绥阳县撤县设区，湄潭县、桐梓县撤县设市，稳妥推进乡镇行政区划调整。五是深化文化体制和旅游综合改革。构建现代公共文化服务体系，建立健全现代文化市场体系。探索建立旅游资源有偿开发利用和旅游资源与旅游景区所有权、管理权、经营权分离等体制机制。六是深化生态文明制度改革。健全自然资源产权管理、用途管制、生态补偿、市场化交易等制度，全面推进赤水河流域“四河四带”生态文明制度改革试点和洛安江流域生态文明先行示范区建设。七是深化国有企业改革。健全国有企业内部管理、内部控制等制度，建立国有资产损失责任追究制度，加快推进国有企业产权制度改革，对符合条件的国有企业进行股份制改造。八是深化开放型经济体制改革。实行外商投资准入前国民待遇加负面清单管理模式（10），不断完善开发区管理机制。九是深化非公有制经济体制改革。建立完善扶持非公有制经济发展的配套政策和落实机制，推进工商注册制度便利化，优化创业环境。十是深化社会事业领域改革。启动机关企事业单位养老保险制度改革，落实县以下机关公务员职务与职级并行制度。全面推进扶贫、就业、教育、卫生、社保等社会领域改革。

实施“十大工程”。计划总投资1460亿元，2015年完成投资443亿元。一是生态文明建设工程。加强生态文明建设和环境治理，积极创建国家生态文明建设示范市。二是“绿智园区”（11）建设工程。推进“1531”产业园区成长工程（12），完善园区基础设施，打造“绿色智造园区”，提升园区建设水平。三是“四在农家.美丽乡村”升级工程。深入推进“6+1”行动计划，打造一批精品示范点。四是“县县通高速”攻坚工程。加快高速公路建设，确保实现县县通高速目标。五是城市交通疏解及“四化”工程。继续推进城市骨干路网和重要节点路口改造，打通城区“微循环”，提升城市绿化、美化、亮化和净化水平。六是文化体育“一场四馆”建设工程。各县（市、区）全面完成1个标准体育运动场和室内体育运动馆、游泳馆、图书馆、文化馆。七是智慧城市（13）创建工程。加强信息化运用推广，促进信息化与工业化、城镇化、农业现代化深度融合，构建“智慧城市”。八是旅游景区精品工程。围绕西线、东线和中心城区环线三条旅游精品线，打造一批旅游精品景区，构建“快进慢游”（14）旅游新业态。九是作风建设升级工程。扎实开展“三严三实”（15）活动，严格落实中央、省、市作风建设有关规定，着力整治机关作风突出问题，提升政府效能和政务服务水平。十是“平安遵义”建设工程。加强社会治理创新，进一步提升群众安全感和满意度，维护社会和谐稳定。

办好“十件民生实事”。计划总投资198亿元。一是完成扶贫生态移民搬迁4660户2.1万人，减少农村贫困人口20万人，实现道真县和20个贫困乡镇“减贫摘帽”。二是新增城镇就业12万人，分别转移和培训农村劳动力10万人、1.5万人；建成90个基层就业和社会保障服务设施。三是建成中心城区中小学校15所，改造全市农村薄弱学校120所、治理地质灾害学校36所，新建、改扩建公办幼儿园150所。四是建成农民体育健身工程140个、全民健身路径工程30个。五是完成15所乡镇卫生院和207个村级卫生计生服务室标准化建设。六是完成城镇保障性安居工程7万套，改造农村危房7万户。七是建成乡镇污水处理厂41个。八是建成中心城区公交汽车停保场，改造中心城区100个社区排污管网和100条小街小巷路灯照明设施，在全市新建100个一类公厕。九是解决70万农村人口饮水安全问题，改造农村电网879公里，建成通村油路1200公里以上，实施退耕还林12.7万亩，治理石漠化197.9平方公里。十是建成城市保供蔬菜基地10万亩。

2015年主要工作任务

一、加快转变发展方式，促进产业转型升级

加快推进新型工业化。坚持全神贯注抓园区、凝神聚气抓工业不动摇，加快工业转型升级步伐，实现工业固定资产投资突破1000亿元、500万元以上规模工业增加值突破1000亿元、规模工业企业突破1000户。着力打造“遵义智造”（16）品牌，推进工业化与信息化深度融合，改造提升传统产业，大力发展以大数据为重点的电子信息产业、新医药大健康产业等新兴产业，做响烟、酒、茶、药、竹和食品“六张名片”，加快推进军民融合发展，加快发展航天航空智能终端设备、可穿戴设备、电子元器件等制造，加强与贵阳、贵安合作推进大数据产业发展，建设“遵义服务外包及呼叫产业示范基地”、中心城区免费Wifi项目，筹办好国际大数据博览峰会、新医药大健康产业集中招商等活动，实现特色轻工产值1000亿元以上、信息产业产值800亿元以上、能源及材料产业产值820亿元以上。指导四大区域主导产业培育，深入实施工业“百千万”工程，及时帮助企业解决生产经营和项目建设难题，大力推进遵义卷烟厂、贵州钢绳集团有限公司等一批重点技改项目建设，加快中电投务川80万吨煤电铝、绥阳循环经济等重点项目建设，建成鸭溪.茅台循环经济产业园、二郎电厂一期等项目，开工建设3000万元以上产业项目500个，建成投产115个以上。加快“绿智园区”建设，强化园区经营理念，积极推进园区投资主体社会化、经营方式市场化、管理模式企业化，完善园区基本功能，建成标准厂房200万平方米、污水处理厂10座、园区道路180公里。培育一批业绩好、带动力强、符合产业政策的企业上市。

加快推进农业现代化。坚持现代农业发展道路，坚定不移实施“1234”战略和“8531”工程，大力发展遵循山地经济规律的现代高效农业，促进一二三产业融合互动，实现农业增加值增长5%、粮食产量265万吨、烤烟155万担以上。强化农业科技支撑，培育壮大特色优势产业，加快35个现代高效农业示范园区建设。着力抓品牌、抓市场销售、抓质量安全，支持无公害、绿色、有机产品认证和茶叶“三绿一红”（17）等重点品牌打造，办好中国.贵州（湄潭）茶博会、上海-遵义农产品展销会等重要农展活动。加大农村土地流转，鼓励适度规模经营，大力培育专业大户、家庭农场、休闲农庄等新型农业经营主体，扶持农业龙头企业发展，新增市级以上龙头企业85家，总量达500家，实现规模农产品加工业产值285亿元。大力实施“321”工程（18）助农增收，加大新型农民培育，提升农业合作社经营水平，加快培育一批集体经济收入过百万元的示范村。全面推进“四在农家.美丽乡村”基础设施建设“6+1”行动计划，加大“三关爱工程”（19）力度，推动“物的新农村”和“人的新农村”（20）建设齐头并进，打造“四在农家.美丽乡村”升级版。

加快发展现代服务业。以承办省第十一届旅发大会为契机，大力发展以文化旅游为龙头的现代服务业。围绕把遵义建成文化旅游创新区和国内一流、世界知名旅游目的地，优化“一心一圈四线”（21）旅游产业空间布局，抓好21个重点省级旅游景区、13个旅游精品小镇建设，新建150个各类精品酒庄、茶庄和特色主题酒店；启动实施仁习赤旅游公路、国家步道、国际自行车赛道建设，打造西部赤水河流域千亿级文化旅游示范带；积极开拓国际、国内市场，推出旅游演艺精品剧目；旅游接待人次和综合收入增长20%以上。大力发展金融、软件和信息技术、现代物流、电子商务等生产性服务业，加快名酒、绿色食品、新材料和现代物流、金融商务会展等综合型服务业集聚区建设。着力推进遵义国际物流城、遵义电子物流园和奥特莱斯、唯一国际等大型物流市场和商业广场建设，积极推进“万村千乡市场工程”（22）和“新网工程”（23）建设，促进物流配送、商贸购物、电子商务发展。大力发展商务娱乐、健康养老、社区家政等生活性服务业，挖掘消费增量。加大消费企业、消费理念培育，促进消费增长，新增限额以上商业企业100家以上。

二、统筹城乡区域发展，促进空间格局优化

统筹四大区域发展。坚持分类指导、分类考核、差异化发展。按照《遵义四大区域生产力布局规划纲要（2014-2020）》，加快区域产业结构优化重组，实现区域优势互补联动发展，提升区域发展实力和水平，县域经济比重提高0.5个百分点以上。中部六县（区）围绕“一主两副两组团一带多园区”（24）空间布局，加快娄山关大道、绥阳至新蒲等城市交通路网建设，实现互联互通同城化发展，提升中部城市综合承载能力；狠抓产业园区发展，做强高速、铁路沿线经济产业带，加快中部率先崛起。地区生产总值、50万元以上固定资产投资、规模工业增加值分别增长14%、25.6%、14%以上。森林覆盖率达49%以上，重点加强100平方公里山体公园建设。红花岗区、汇川区、遵义县、桐梓县力争进入全省经济强县前10、前15、前7、前20位，绥阳县力争挤进全省经济强县。西部三县（市）全面推进“四河四带”建设，主抓酱香白酒、优质煤炭、生态旅游、红色文化，凸显中国酒都、绿洲红城、丹霞竹海等区域发展特色，增强西部经济的拉动作用。地区生产总值、50万元以上固定资产投资、规模工业增加值分别增长14.5%、27.5%、13.5%以上。森林覆盖率达58%以上，重点抓好赤水河沿线景观打造。仁怀市继续保持全国百强县（市）地位，力争赤水市进入全省经济强县前15名、习水县进入非经济强县前6位。东部三县围绕农村美、农业强、农民富，立足已有优势提档升级，探索联动发展，加快推进“四在农家.美丽乡村”生态文明示范区建设，围绕“一江一湖一海”（25），联合打造全国知名、贵州一流的生态休闲旅游示范区。地区生产总值、50万元以上固定资产投资、规模工业增加值分别增长15%、28.6%、15%以上。森林覆盖率达55%以上，重点加强景观打造和植树造林。湄潭县、余庆县、凤冈县力争进入全省非经济强县前3、前5、前20位。北部三县按照“三心三园两带多点”（26）空间布局，抓好正安瑞新、道真上玉和务川煤电铝三大园区，强化芙蓉江和洪渡河沿线产业带发展，稳定烤烟、中药材等经济作物生产，进一步加大项目倾斜和产业扶持，确保北部减贫人口7万人以上。地区生产总值、50万元以上固定资产投资、规模工业增加值分别增长15%、28.7%、16%以上。森林覆盖率达56%以上，重点加快退耕还林还草建设。正安力争进入全省非经济强县前15位，道真、务川力争排名提升8位以上。

推进山地特色新型城镇化。坚持新型城镇化发展方向，推进以中心城区为龙头、节点县城为骨干、示范小城镇为补充的黔北城市群建设，筑牢承载200万人口的中心城区发展基础，完成投资1400亿元，城镇化率达45%以上。坚持规划引领，推进“多规融合”，严格落实“五线”控制（27）和“四先四后”（28），在城市规划建设中突出传统文化、自然生态和历史遗迹保护。坚持以产兴城、以城促产，加快中心城区“3个100平方公里”（29）建设，围绕21个城市综合体和54个市级以上示范小城镇，打造城市建设新引擎和城乡统筹融合点。落实建筑业发展扶持政策，加快建筑业转型升级，大力培育发展新型建筑和建材业，建筑业总产值增长40%以上。规范房地产市场秩序，促进房地产业健康有序发展。加大城市基础设施、教育医疗卫生等公共配套设施建设，推进户籍制度改革，加速农业转移人口市民化和人的城镇化。加强城市管理，进一步理顺城管执法体制，提高城市管理综合执法水平，坚决打击违法建设；启动不动产权登记，规范物业管理运行，建立完善网格化、精细化、信息化管理体系。推进新蒲、南白、绥阳等城市建成区公交全覆盖。统筹“智慧城市”、“公交都市”创建，不断增强城市活力，彰显城市魅力。

三、实施基础设施攻坚，促进发展条件改善

实施交通建设攻坚。大力推进交通三年会战，完成交通投资 260亿元。务正、道安高速全线建成通车，加快贵遵复线、江安、务德、江习古、白腊坎至黔西高速建设，开工建设绕城西南环、绥正、习正高速，高速公路通车总里程突破1000公里。改造国省干线200公里以上，公路通车总里程突破3万公里，实现县县通高速、村村通油路目标。加快渝黔快铁、川黔铁路外迁、阁老坝货场等项目建设，力争开工中心城区轨道交通一期工程，加快贵阳至遵义城际铁路、攀昭黔铁路等前期工作。遵义机场年旅客吞吐量力争突破100万人次，争取仁怀机场建成试航。完成构皮滩水电站翻坝运输系统，加快湄江、洪渡河、芙蓉江等库区航运工程建设，力争开工赤水河和乌江渡库区航运扩能工程。

实施水利建设攻坚。大力推进水利建设三大会战，完成水利投资60亿元。开工建设遵义绿塘等5座中型水库和凤冈崇新等8座小型水库。完成25座新增病险水库治理。加快余庆等小农水重点县项目建设，发展农村耕地保灌面积15.5万亩。

实施电力通讯攻坚。加快火电、水电、风电开发，电力总装机突破900万千瓦。实施农网改造升级工程，建设6个220千伏、14个110千伏、7个35千伏输变电工程。实施信息基础设施三年会战，新增光缆线路3.2万公里、3G/4G基站2900个，覆盖家庭53万户；实施城市有线电视双向化改造8.4万户，新增农村数字电视用户5万户。中心城区无线网络、行政村通宽带实现全覆盖。

四、加强生态环境保护，促进生态文明建设

推动国家生态文明建设示范市创建。坚持生态第一要求，巩固“创模”成果，积极推进国家生态文明建设示范县、示范乡镇创建，为创建国家生态文明建设示范市奠定基础；积极发展生态经济、培育生态文化，完善生态文明考核评价体系。倡导绿色出行、绿色建筑等低碳生活方式，积极构建现代文明生态文化体系。推动湄潭、赤水创建国家生态县（市），确保新增25个国家级、64个省级生态乡镇。

加强生态建设和环境治理。完成营造林75.6万亩，森林覆盖率提高到54.7%，治理水土流失350平方公里、中小河流18条。强化城乡垃圾收储设施建设、农业面源污染防治、工业渣场和尾矿库治理。完善空气质量监测和重点污染源在线监控预警系统，完成中心城区主体功能区红线划定。坚持重典治乱、铁拳铁规治污，深入开展“六个一律”环保“利剑”专项行动（30）。中心城区空气质量优良率（六参数（31））达80%以上，县城以上和乡镇集中式饮用水源地水质达标率分别达100%、95%以上，城镇污水集中处理率、城乡生活垃圾无害化处理率分别达85%、80%以上。

强化资源能源节约利用。坚持进退并举、优胜劣汰，大力淘汰落后产能。加快中心城区重点企业退城进园、异地技改。推进国家新能源示范城市、国家低碳城市、资源节约型环境友好型企业等创建，打造生态工业示范园区。加强粉煤灰、酒糟、赤泥等工业废弃物和污水综合利用，工业固体废弃物综合利用率66%以上，工业用水重复利用率80%以上。推进土地节约集约利用，严守耕地红线，加强1000亩以上坝区耕地保护，做好城乡建设用地增减挂钩，确保耕地保有量、基本农田保有量不低于省下达指标。

五、强力推进开放创新，促进发展活力释放

强力推进对外开放。坚持开放带动，积极融入“一带一路”（32）和长江经济带（33）建设。主动承接上海对口帮扶，力争沪遵在教育、医疗、人才、科技和产业转移、园区共建等方面取得新突破。加强与泛珠三角、成渝经济区等区域合作，深化同战略合作城市、企业集团、金融机构和科研院所交流与合作，力争取得新成果。加大开放平台建设，开放遵义机场航空口岸，力争遵义综合保税区申建取得突破性进展，支持海关、检验检疫、外汇管理等设立分支机构。积极培育外经贸市场主体，新增外贸企业50家、外资企业10家以上。力争实现进出口总额、利用外资增长20%以上。加大招商引资力度，引进500强企业和行业龙头企业20个以上；引进省外到位资金1950亿元，增长23.5%以上。

强力推进创新发展。坚持创新驱动，深入推进国家创新型试点城市建设，大力实施科技管理改革、科技开放合作、科技企业培育、科技金融融合、科技服务能力提升“五大行动”，围绕重点产业、重点领域建立和完善科技创新体系，突出企业创新主体地位，加强科技交流与合作，鼓励产学研合作，培育多层次科技创新平台和科技创新人才团队，建设一批工程技术研究中心、重点实验室、院士（专家）工作站、科技企业孵化器，推动科技研发和成果转化，加速产业转型升级。推动大学科技创新产业园规划建设，新增各类创新平台20个，累计高新技术企业达到80户。大力实施知识产权战略，发明专利申请量突破1000件。深入实施“百千万人才计划”和“15851”人才精英工程，加快遵义人才城和人才服务体系建设，用好高校资源，加大人才培养力度，引进高层次人才500名以上、各类急需紧缺人才8500名以上。

促进民营经济发展。落实民营经济五年提升行动计划，扎实开展服务环境整治、产业升级优化、“双增”示范提升、民营企业培育、民企产品市场开拓、服务体系升级、引贤育才“七大工程”，建立14个县级中小企业服务中心和19个产业园区服务中心，形成一体化服务网络。大力实施中小企业成长和万户小老板创业工程，充分激发各类市场主体创业热情，新增市场主体5万户，扶持微型企业1900户以上。民营经济完成增加值1130亿元，比重达54%以上。

六、全力保障和改善民生，促进全面小康创建

大力推动社会事业发展。全面推进基本普及十五年教育，深入实施教育“9+3”计划。学前三年毛入园率83.5%，义务教育巩固率90%，高中阶段毛入学率85%。加快遵义大学城、遵义职教园区建设，建成投用遵义医学院二期、遵义职院二期、遵义师院一期、遵义干部学院，力争遵义大学创建取得突破性进展，启动贵州（湄潭）茶学院建设。建成正安、凤冈、余庆三个县级综合档案馆。大力培育和践行社会主义核心价值观，着力加强传统文化保护，弘扬传承中华文明。力争海龙屯成功申遗，启动茅台镇“双遗产”（34）申报工作。深化医药卫生体制改革，完善医疗卫生服务体系，提升基本公共卫生服务均等化水平。建立健全市县乡村四级食品药品监管体系，推进重大疾病预防控制体系建设试点工作，全面加强卫生安全监管工作。深入推进全国文明城市创建，巩固国家卫生城市创建成果，确保赤水市、余庆县、遵义县成功创建国家卫生城市（县城）。围绕“争一保二”目标，全力备战全省九运会。全面推进民族宗教、外事侨务、人民防空、国防教育、机构编制、血液安全、气象水文、妇女儿童、残疾人、地方志、红十字等工作。

大力提高社会保障水平。全面落实就业、扶贫、住房保障等政策，推进城乡低保、医疗救助、特困人员供养等社会救助体系建设，支持慈善事业发展，筑牢社会保障网。城镇医保参保131万人，新农合参保率95%以上，城乡居民养老保险参保296万人，城乡低保提标10%以上。统筹加强灾害救助、殡葬改革等工作。建立多元化养老服务体系，新增养老床位7000张以上。

大力创新社会治理方式。落实维护稳定第一责任，依法严厉打击各类违法犯罪活动，加快社会治安立体化防控体系和犯罪适时控制机制建设，强化灾害防御、应急管理、公共安全监管、特殊人群服务工作，推行网格化服务管理，确保群众安全感和满意度保持在全省第一方阵。深入推进“法治遵义”建设，不断深化法律服务和法律援助工作。扎实推进送法律、送政策、送温暖，党群干群心连心“三送一连心”民心工程和“四民社区.美丽城市”创建。加大矛盾纠纷排查化解，落实社会稳定风险评估和重点信访案件领导包案化解制度。坚持依法治安，全面推进安全生产标准化建设，落实安全生产责任制，杜绝重特大事故，严防较大事故，控制一般事故。

七、加快建设法治政府，促进政府效能提升

全面依法履行职责。坚持职责法定原则，进一步清理和规范权力运行流程，并向社会公开，严禁法定审批流程外擅自增加环节、附加条件。全面推行政府权力清单、责任清单、服务清单有机统一。坚决查处不作为、慢作为、乱作为行为。健全完善依法决策机制，把公众参与、专家论证、风险评估、合法性审查和集体讨论决定作为重大行政决策的法定程序，重大事项决策先经政府集体研究，报请同级党委或人大审议同意后执行。完善行政决策监督问责机制，落实领导干部决策批示备案和责任追究制度。全面深化行政执法体制改革，整合行政执法力量，推进执法重心向基层下移，积极探索城市管理等重点领域综合执法。严格行政执法资格管理，严禁未取得执法资格人员执法。严格执行罚缴分离和收支两条线管理制度；严格规范公正文明执法，认真落实行政裁量权基准制度（35），确保依法裁量、精准执法。全面推行机关事业单位法律顾问制度。充分发挥行政复议化解行政争议的主渠道作用，提升行政复议公信力。

倡导全民学法用法。坚持把法制教育纳入国民教育体系、精神文明创建和干部职工学习内容。落实“谁执法、谁普法”责任制，深入推进“六五”普法和“法律八进”（36）活动，办好法制宣传栏目，加强法治公园、广场、长廊等普法场所建设，开展群众性法治文化活动。坚持依法治市和以德治市相结合，健全公民和组织守法诚信记录，完善守法诚信褒奖机制和违法失信惩戒机制，引导人们自觉履行法定义务。健全法律服务体系，完善市、县、乡、村、组五级法律服务网络，免费为公众提供法制宣传、法律咨询、法律援助、纠纷调处等公共法律服务。坚持依法解决纠纷原则，完善信访工作机制，促进依法信访，引导全民自觉守法、遇事找法、解决问题靠法。

自觉强化各方监督。主动接受人大法律监督、政协民主监督，认真办理人大代表建议、政协委员提案，高度重视民主党派、工商联、无党派人士意见建议。强化行政监察，切实加强政府内部权力制约。强化审计监督，保障依法独立行使审计监督权，加大审计成果运用。强化社会监督，完善群众举报受理制度，设立投诉网站、举报邮箱、举报电话等公众直通交流平台，畅通群众监督渠道。强化舆论监督，对新闻媒体反映的问题，及时调查核实，依法作出处理并公布结果。强化公众监督，坚持公开为常态、不公开为例外，推进决策公开、执行公开、管理公开、服务公开、结果公开。完善政府新闻发言人制度，及时回应群众关切。

各位代表！作风建设永远在路上。我们将全面巩固党的群众路线教育实践活动成果，持续推进机关作风建设，严格执行中央“八项规定”、省委“十严禁”“十不准”等各项规定，全面推进机关内部和市直机关之间科长交流轮岗制度，重拳整治“中梗阻”等作风突出问题，不断优化招商引资环境，提高服务水平。坚持厉行节约、反对浪费，大力营造风清气正、务实高效、勤政廉洁的政务环境。坚持干字当头、立说立行，大力弘扬钉钉子精神，建立目标倒逼机制，强化督查督办和行政问责，全面增强政府执行力和公信力。严格落实党风廉政建设主体责任，切实履行“一岗双责”，推进政府廉政建设和反腐败工作取得新成效。

各位代表！目标已确定，发展凭实干。让我们紧密团结在以习近平同志为总书记的党中央周围，在省委、省政府和市委的坚强领导下，团结一致、克难攻坚，行稳致远、奋勇向前，全面完成“十二五”规划目标，不断把后发赶超、全面小康伟大事业推向前进！
